# To summarise the approach to and findings of the PPIE undertaken as part of a programme of secondary research with a vulnerable, hard to reach population during the COVID-19 pandemic

**DOI:** 10.1186/s40900-023-00416-7

**Published:** 2023-05-10

**Authors:** Niall McGrane, Paul Dunbar, Laura M. Keyes

**Affiliations:** Health Information and Quality Authority (HIQA), Unit 1301, City Gate, Mahon, Cork, Ireland

**Keywords:** Patient public involvement and engagement, COVID-19, Residential care facilities, Nursing homes, Secondary analysis project

## Abstract

**Background:**

Public and patient involvement and engagement (PPIE) is an important part of research. The inclusion of PPIE in research is becoming more widespread, however, there are some areas where it is still uncommon. For example, undertaking PPIE in secondary analysis projects is uncommon and PPIE with difficult to reach populations and vulnerable groups can be seen as being too difficult to facilitate. The aim was to summarise the approach to and findings of the PPIE undertaken as part of a programme of secondary analysis with a vulnerable, hard to reach population; residents of residential care facilities (RCFs), during the COVID-19 pandemic.

**Methods:**

As part of a project to develop a publically available database of statutory notifications of adverse events from RCFs in Ireland, residents (n = 9) from RCFs for older people and people with disability were telephone interviewed. Residents were engaged through gatekeepers and posted participant information and consent forms. Themes were identified using content analyses of interview notes.

**Results:**

Three parent themes were identified, each with two subthemes: privacy concerns, enthusiasm and dissemination of research findings. Residents highlighted the importance that no personal information be shared in the database. Once data were anonymized, residents thought that the database should be published and shared. Residents reported being happy about research being undertaken using the data and thought that publishing the database would help inform the public about RCFs. Completing a PPIE project with a vulnerable group during the global COVID-19 pandemic required planning and resources. Resources included finances, time and expertise.

**Conclusions:**

The involvement of residents informed the data inclusion in the published database and the approach taken in the protection of personal data. Enthusiasm for publication and research using the database by residents encouraged the developers as it was considered something that was wanted by residents. The benefits of PPIE can be achieved with vulnerable groups during unprecedented times with the appropriate planning. It requires dedication of time, finances and expertise. Overcoming the obstacles was achievable and worthwhile. The approach outlined can be used as an example to support PPIE in secondary analysis projects and or with vulnerable groups.

**Supplementary Information:**

The online version contains supplementary material available at 10.1186/s40900-023-00416-7.

## Plain Language summary

When doing research, it is important to involve the people that the research is going to impact. Research projects are increasingly including this involvement, however, there are some areas of research where it is not yet commonplace. For example, when undertaking projects with existing data or when the people of interest are considered a difficult to reach or vulnerable group. The LENS project (LEarning from Notifications in Social care) is a research project that developed and published a database using existing data on safety incidents in care homes. We carried out a consultation with people living in care homes as part of this project. People were asked for their input on the development of the database and the research that was being carried out on it. This work was completed during the COVID-19 pandemic and, as such, the people giving input were considered a vulnerable group. The people living in care homes were concerned about personal details being included in the database. Once they were reassured that no personal information would be included they were supportive of the database being made public. They also offered ideas about how the research that used the database could teach people working and living in care homes. The article describes how the researchers engaged the people and what was needed to achieve this. The article shows that involving people from vulnerable groups and in research relating to existing data, adds value even though there are obstacles to overcome. It is summarised here as an example for researchers undertaking similar work.

**Supplementary Information:**

The online version contains supplementary material available at 10.1186/s40900-023-00416-7.

## Introduction

Public and patient involvement and engagement (PPIE) is an important part of health and social care research, in recent years it has been heavily promoted and encouraged and is now routinely part of applications for research funding and summaries of PPIE activities are specifically called for by some academic journals. PPIE has been defined as research carried out ‘with’ or ‘by’ members of the public rather than ‘to’, ‘about’ or ‘for’ them [1]. This means collaborating or partnering with patients, service users, carers, families using health and social care services, people with lived experience of health conditions, patient advocacy organisations, and members of the public. PPIE has a role in most aspects of healthcare research projects, in identifying and prioritising areas of importance, in designing and managing projects, in analysing and interpreting data, in dissemination and in implementation of findings [1].

Involving patients or members of the public in secondary analysis projects is uncommon. Some of the more traditional roles of PPIE, recruiting and managing participants for example, are not applicable and there is no accepted means by which to facilitate PPIE in secondary analysis research. The approach taken for PPIE, however, does not have to be standardised and PPIE can have positive impacts on all stages of research [2, 3].

PPIE is undertaken with the general public or in some cases with specific patient groups. There are, however, more difficult to reach populations and vulnerable groups that research should be carried out with, and by, rather than for, or about. PPIE can be seen as being too difficult to facilitate with members of these groups. They should not, however, be disenfranchised or overlooked during research. Living with, for example, an intellectual disability, does not prevent valuable contributions to research [4–6]. Involving members of these groups, is fundamental to gaining insights to their lived experience however there are specific considerations to be resourced and planned for when involving members of vulnerable groups [4, 5].

This paper, therefore, aims to summarise the approach to and findings of the PPIE undertaken as part of a programme of secondary analysis with a vulnerable, hard to reach population; residents of residential care facilities (RCFs) during the COVID-19 pandemic.

## Methods

### Scenario description

RCFs provide accommodation, care and supportive services to people who cannot live independently. They encompass nursing and residential homes, supportive care facilities, rehabilitation and palliative care centres, amongst other care facilities. RCFs provide different levels of care to a range of people, from full nursing care to assisted living and from respite to full-time care.

Internationally, residential care facilities (RCFs) are typically regulated to promote quality and safeguarding. A common feature of regulation is the statutory responsibility of RCFs to notify the regulator about adverse events (AE) [7–12]. In RCFs, the interpretation of AEs is typically broader than acute settings and applies to events that have potential or actual impact on the quality and safety of care and wellbeing of residents**.** Examples of AEs in RCFs include but are not limited to allegations of abuse, serious injury, unexpected deaths, staff misconduct and loss of a service such as power or water. The prevention of AEs in health and social care services remains a challenge.

The LENS project (LEarning from Notifications in Social care) was formed to address this challenge. It is a secondary data analysis project of previously collected data on notifications of AEs. The LENS project aimed to develop a publicly available, analysable database of notifications of adverse events received by the social care services regulator in Ireland. Upon completion and publication of the database, various secondary analysis studies utilising the database were undertaken as part of the LENS project.

The database developed, known as the Database of Statutory Notifications from Social Care in Ireland [13], contains data surrounding AEs involving vulnerable populations, older people and people with disability. While the LENS project does not have participants, members of these groups who are residents of RCFs may be impacted by any improvements in the quality of care as a result of the LENS project research. The LENS project is examining data on events but it is the residents of RCFs whose opinions on this research matters, the LENS project is researching ‘for’ these people. The aim of the PPIE aspect of the LENS project was to involve and gain input from people living in RCFs on the design, development and publication of the Database of Statutory Notifications from Social Care in Ireland. Input was also sought on research carried out by the LENS project on the database, interpretation of findings and dissemination.

The COVID-19 pandemic arrived in Ireland just as the LENS project began. Therefore the public health restrictions that were introduced were accounted for in the methodology of this PPIE.

### Ethics

Ethical approval was sought for this PPIE study as it involved a vulnerable population group. Ethical approval to engage with people with disability living in residential care facilities for PPIE purposes was granted by the Research Ethics Subcommittee of the Daughters of Charity Disability Support Service Ethics Committee on 26/01/21. Ethical approval was obtained from the Tallaght University Hospital/St. James’s Hospital Joint Research Ethics Committee (29/06/2021) to engage with older persons living in RCFs for PPIE purposes.

### Study design

The PPIE was conducted and reported according to the Guidance for Reporting Involvement of Patients and the Public (GRIPP2) [14] (Additional file 1) and the Standards for Reporting Qualitative Research (SRQR) [15]. Semi-structured interviews were used to collect insights and opinions and were analysed thematically.

### Sample

The health and social care regulator in Ireland is responsible for registering and inspecting residential services for older people and people with a disability. Permission was granted by the regulator, a collaborator and knowledge user for the wider research programme, for the research team to contact RCFs to conduct interviews. Ireland had a total of 567 RCFs for older people and 1401 for people with disability at the end of 2021 with each assigned to an inspector for regulation and monitoring purposes. One RCF for older people and one RCF provider (consisting of several houses) for people with disability were selected based on largest bed numbers to maximise recruitment potential and to support effective use of researcher time and resources.

### Recruitment

The inspector responsible for the selected RCFs introduced the researchers to the person in charge, the appropriately qualified manager of the RCF, by way of email and phone contact.

The researchers provided the person in charge with the study information leaflets and the informed consent forms and answered any questions they had regarding the study. The person in charge introduced the study to residents and asked them if they wished to participate. Inclusion criteria included having capacity to provide informed consent and ability to sit through an interview lasting 30 min to an hour. Residents who expressed an interest were provided with a participant information leaflet and a consent form from the person in charge. Signed informed consent forms and phone numbers were emailed to the interviewer. Residents were afforded the opportunity to ask questions prior to beginning the interview and were informed that they could withdraw their consent at any time and that they could any family or a member of staff to assist them with the interview.

### Data collection

Once residents consented to an interview, a date, a time and a preferred method of interview (telephone or video-call) convenient for the resident was agreed upon. Prior to beginning the interview the resident and any family or support staff assisting the resident were offered the opportunity to ask questions regarding the study and the interview. The resident was notified that they could request a break from the interview and again notified that they could withdraw their consent at any time.

The interviews followed a schedule of questions (Additional file 2) and were conducted by one researcher who was experienced in conducting research interviews. Key points and quotes were recorded. Opinions and insights into two different topics were sought during the interview. The first pertained to the development of the Database of Statutory Notifications from Social Care in Ireland [13]. The second pertained to research being carried out by the researchers using this same database, in general and specifically on practice surrounding adverse events in RCFS in Ireland [16]. No demographic data, other than sex, were collected as we felt it was unnecessarily intrusive in a study of this nature as demographic data would not impact on the results of the study. A person’s sex, however can impact on a person’s experience of care [17]. Upon completions of the interview, the interviewer summarised and paraphrased the conversation, repeating it back to the resident to validate the results. The residents were then provided with the opportunity to amend, dispute, add or withdraw anything.

### Data management

All interview data were anonymised. Residents’ contact details were securely destroyed immediately following the completion of the interview phase of the study. All anonymised interview data were stored on an encrypted and password protected network, accessible only to the research team. The data will be retained for one year following the completions of the wider programme of research.

### Data analysis

A Qualitative Descriptive (QD) approach was applied [18–20]. The six phases of thematic analysis as described by Braun and Clark [21] were carried out independently by two researchers who met and discussed findings upon completion of each phase to ensure consistency and agreement of interpretations. Interviews were conducted until saturation of themes was reached and agreed upon by 2 researchers based on iterative review of interview notes. A coding tree was agreed upon and quotes were used to support a narrative summary. Each quote was attributed to the resident using their study ID, which was placed in square brackets.

Both researchers were experienced in thematic analysis as described by Braun and Clark [21]. Both reviewers were experienced qualitative researchers with experience of working as healthcare professionals with older people and people with disability. The first reviewer carried out the interviews and then shared the notes and discussed each interview with the second reviewer.

## Results

### Sample

A total of nine residents (seven female and two male) were interviewed. Three out of these nine are residents of RCFs for older people (two female and one male). All three of these interviews were conducted by telephone with no assistance from family or members of staff. Six out of the nine are residents of RCFs for people with disability (one male and five females). Of these six, one was interviewed by video call and the rest were interviewed by telephone. Of these six, three had a member of staff on the call with them to assist with the interview.

### Themes

**Fig. 1:**
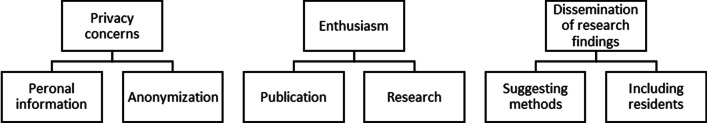
Themes and subthemes

Three parent themes were identified, each with two subthemes: privacy concerns, enthusiasm and dissemination of research findings (Fig. 1). All themes and subthemes were identified from interviews with residents of RCFs for disability and of RCFs for older people.

Residents discussed privacy in two ways, the first was their concern that the database contain no personal information; “No personal information, names etc. Anything that could be used to bully someone” [LENS01]. “No personal information, anything like GDPR. Privacy is very important” [LENS06]. The residents did not want personal identifiable information contained in a database that would be made available to the public. The second subtheme in privacy was anonymization. Residents thought that once data were anonymized that the database should be made available; “I’m happy that this [anonymized data] is available” [LENS03]. “I’m happy that all personal data is removed and no individual or centre can be identified” [LENS07]. Anonymization of the database reassured the residents that individuals and RCFs would not be identifiable from the database. All residents agreed with the intention to publish the data: “Yes very, everyone’s voice should be heard” [LENS01].

Residents were enthusiastic, firstly about the publication of the Database, making it available to researchers and the public; “It’s [publishing the database] a great idea, I can’t think of any negatives to it at all” [LENS08], “super idea that they [researchers and the public] have access [to the database]” [LENS07] “It‘s a good thing that can help people and how they live.” [LENS01]. Secondly, residents were enthusiastic about publication of research on management surrounding AEs; “Yes it’s a good idea to share the good work being done in the nursing home.” [LENS07]. Residents thought publishing would help inform the public about RCFs, how residents live and the good work that is ongoing in RCFs**:** “We should be telling the public. The public don’t understand how people with disability live in the community.” [LENS01], “Good idea to tell people about the good and the bad.” [LENS06].

The final theme was dissemination. Residents suggested different methods for disseminating results of a study on management surrounding adverse events in RCFs; “Good practice guides and training days a good idea” [LENS07]. “Include pictures and videos. Use Zoom and IPads” [LENS04], “People can learn in groups and individually, [learning] from someone close to you is a good idea.” [LENS05]. The second subtheme addressed residents’ opinion that residents themselves should be included in any planned learning of the research carried out by the LENS project; “We should include the clients in this. There is learning for them as well” [LENS01], “Residents could learn about it during their activities” [LENS08].

Residents were asked their opinion on whether the current list of incidents that require reporting was appropriate and if any additions were to be made. All residents agreed that the regulator should be notified of each event. Some residents made suggestions, such as bullying, that come under the remit of an existing notifications. No new events were identified by residents.

### Resources

This study took place during the COVID-19 pandemic where residential care facilities were particularly vulnerable to outbreaks of infection. It was for this reason that in-person interviews or in-person focus groups were dismissed in favour of telephone and video calls. This choice resulted in a cost saving as telecommunication required no travel or hosting expenses. Challenges remained, however, due to the pandemic. Coordinating interviews with residents and staff was difficult but not insurmountable. RCFs were dealing with outbreaks of COVID-19 among residents and among staff during this period often making staff and residents unavailable for interview. The data collection phase of this PPIE was completed over a three month period.

Interviews took between 15 min and half an hour to complete. Three residents, all from RCFs for people with disability, had members of staff assisting with the interview. Assistance consisted of re-phrasing or repeating questions for residents and repeating resident’s answers. This assistance aided clarity of questions and answers. Key points and quotes were recorded in lieu of recording and transcription to aid time efficiency.

Thematic analysis was completed by two researchers in one month period, with one researcher dedicating 25% FTE to the PPIE and the second researcher dedicating 5% FTE. Choosing appropriate RCFs and completing their ethic applications took one researcher one month, dedicating 25% FTE. Ethics committee approval took a further 2 months and 5 months, respectively.

## Discussion

PPIE has been defined as research carried out ‘with’ or ‘by’ members of the public rather than ‘to’, ‘about’ or ‘for’ them [1]. Secondary analysis projects are more akin to research being carried out “about” or “for” people as they do not, by their nature, involve direct patient or public involvement. In this PPIE, research was carried out “with” residents as they advised, criticised and recommended changes and improvements to different aspects of the project. PPIE in research improves the quality of research projects, improving their application and impact. These positive outcomes are reported during all stages of the project, from design to dissemination [3, 17]. These benefits of PPIE are still valid when conducting research with vulnerable groups despite the perceptions that research with these groups can be too difficult to facilitate [4–6]. PPIE does not just happen, it involves planning and resources. There are practical aspects to PPIE, planning, collaborating with groups, managing participants and analysing data [3]. These must occur prior to and throughout the process to maximise and optimise input and outcomes and can be timely and costly [3]. These same challenges are still present, or heightened, when conducting PPIE with vulnerable groups but this research, in addition to other PPIE projects with vulnerable groups, confirm that people with a disability, or similar, can effectively contribute to research projects.

### Summary of PPIE findings

As the LENS Project is a secondary data analysis project some of the usual areas PPIE can contribute to were not applicable. The LENS project does not have participants, residents of RCFs are not directly involved in the study and the data contained in the Database of Statuary Notification pertains to events, not individuals. There were, however, valuable contributions to the LENS project made by residents of RCFs. Contributions were made to the design, dissemination and knowledge translation plans.

Three parent themes were identified and there was evidence for these in each interview, from both settings. Privacy was a major concern of the residents, reiterating the concerns of the LENS project team who were already in the process of ensuring the database did not contain any information that could be used to identify an RCF or an individual [13]. The concerns of residents reinforced the focus of anonymity by the team. Once residents were reassured that the database would not contain any personal identifiable information they expressed enthusiasm for publication of the database and its use by researchers other than the LENS project. They also expressed enthusiasm for the publication of the secondary data analysis research. Residents viewed the LENS project as a good thing for residents of RCFs. Residents addressed dissemination making suggestions on how best to implement the findings of research. Residents suggested including residents in already planned for training and learning based on the LENS project findings. As a result an infographic for RCFs, aimed at residents, based on LENS project work was planned for. Residents of RCFs agreed with the interpretation of the findings of the LENS study on management of incidents in RCF, which strengthens these findings.

### Reflections on approach

During the initial planning of the PPIE work there was no global pandemic and no public health restrictions. The restrictions imposed in response to COVID-19 caused severe disruption to PPIE [22, 23]. RCFs applied restrictions, prohibiting visitors, which made in-person engagement impossible. This disruption resulted in a change in methodology but did not alter the aims and objectives. The goals of this PPIE work were achieved despite these disruptions by utilising telecommunications. Restricting visitors was not the only disruption. Interviews were conducted after a year of dealing with the implications of a pandemic and had to be organised and coordinated with RCFs who were dealing with outbreaks of COVID-19 among residents and staff, stretching resources, which had been stretched for over a year.

In-person focus groups may have been more beneficial to foster conversation and debate among residents but was rendered impossible due to the COVID-19 pandemic. In addition, it has been reported that PPIE focus groups can included users influencing each other and groups being dominated by individuals, their perspectives or their personal experiences [3]. Online focus groups where considered as an alternative but were dismissed. Coordination of staff and residents online was deemed too difficult due in part to the effects of the pandemic on staffing. Individual interviews, however, gave residents privacy to discuss their own personal opinions without the influence of others. Individual interviews provided residents the opportunity to have a known support person with them, which could not have been facilitated in a focus group setting. Individual interviews therefore offered residents the opportunity to converse with the researcher in privacy and comfort which facilitated more in depth conversation and honest opinions. This was the most appropriate methodology when engaging with residents of RCFs despite the extra planning and resources involved. More time was spent organising individual interviews with individual residents as opposed to organising two focus groups. The interview schedule was updated with additional questions if new topics were discussed by residents.

Individual interviews were conducted using telecommunications due to visitor restrictions placed on RCFS due to the COVID-19 pandemic, to which residents of RCFs were particularly vulnerable. Telecommunications enabled planning of interviews to be flexible in terms of time, environment and location. Residents did not have to travel and could speak with the interviewer in a location of their choice, in the comfort and safety of their own home. Telecommunication is however limited as it does not include non-verbal communication and there can be poor audio on calls. Residents were offered the opportunity to have someone assist them with the interview, this improved understanding and clarity of questions and answers in cases of poor quality of audio and enunciation. All residents had access to the necessary technology, however this may not be the case for all potential PPIE participants and may have limited who could take part. Comparable benefits and limitations have been reported in similar PPIE projects conducted during the COVID-19 pandemic [18]. The approach taken for this PPIE was very effective while using fewer resources, taking less time and costing less financially. The input from residents was no less valuable with this method while there was no travel expense and no travel time in addition to no focus group organisation and hosting for RCFs.

Residents of RCFs for older people and people with disability are members of vulnerable groups, therefore specific requirements were necessary for this study as has been reported in the literature [3–6]. These additional considerations include extra time and therefore costs, communication and consent, and recruiting and ethical requirements. Not all PPIE requires ethical approval but as we were involving members of vulnerable groups, we sought ethical approval for this work. Ethics applications require resources, and time. Ethics applications had to clarify how this study could be conducted with a vulnerable group during a pandemic to which residents of RCFs were particularly vulnerable. Other considerations were accounted for with this group; staff and or family could be present if the resident wished. This ensured effective communication as those familiar with residents could clarify responses if necessary. The time taken for ethical approval will vary depending on numerous factors beyond the control of researchers. Lengthy ethical approval procedures will however be seen as a barrier to including members of vulnerable groups in PPIE, especially projects operating with short timelines.

The collection of sociodemographic data on residents is almost universal, however it is best practice to only collect relevant data that addresses the aims of a study. Hence, sociodemographic details on residents, reason for residing in an RCF for example, were not collected as these data was not necessary as it would not contribute to the findings. Resident contact details were destroyed upon completion of the interviews and insight data will not be kept for a prolonged period. As the LENS project is secondary analysis there was no need to re-contact residents for later stages of the research as may be the case with primary research projects. Residents were not remunerated for their time. However, upon reflection on good practice, a reward for sharing their time and expertise would have been appropriate.

The sample size of 9 was small with only 3 residents of RCFs for older people partaking. The RCFs chosen were located in urban areas, the reason for this was to limit travel and the associated expenses. This however, became irrelevant due to the COVID-19 pandemic. The small number of urban dwelling residents may have limited the collection of different opinions, however, saturation of themes was reached. Following a schedule of questions aided this. The RCFs where residents lived varied in size, from small households with less than 5 residents to a large nursing home. Input from residents of rural RCFs and those in smaller RCFs may have offered different opinions on privacy given that they may be potentially easier to identify. This was a concern of the authors and was addressed during the development of the database [13]. Further input from more people and from rural settings could have been sought but would entailed additional ethics applications, taken more time and would have consumed additional resources from the research team and from the RCFs who facilitated the interviews. In light of the COVID-19 pandemic, RCF’s finite resources and that saturation had been achieved no further interviews were held.

Limiting participation to individuals who could provide informed consent and partake in an interview excludes those residents who can’t. As RCFs are homes to many people with a range of abilities our inclusion criteria excluded the opinions of those who may customarily not have their opinions heard. This may be a reason a smaller number of residents from RCFs for older people were recruited as residents of these facilities who meet the inclusion criteria are in the minority. Our sample, therefore, is likely not representative of all residents of RCFs, however a range of views was obtained and saturation was achieved. Recruitment of individuals who cannot provide consent or tolerate an interview was not feasible for the LENS project as it would require additional ethical considerations, additional time, additional resources and different methods, such as individualised communication [24] or photo-elicitation interviews [25]. Future research with residents of RCFs should account for and plan for the extra resources, addition methods and ethical considerations necessary to include all individuals.

The LENS project researchers are employed by the regulator and this may have introduced a bias or be seen as a conflict. Every effort was made to mitigate against this. The LENS project is externally funded and independent from the regulator, the RCFs acted as gate keepers and the researcher who conducted the study and interviewed the residents works exclusively for the LENS project and has no other role within the regulator. This was made clear to the RCFs and the residents verbally and in written format prior to the beginning of the study. Some residents did however take the opportunity of the interview to enquire if the regulator could intervene in personal matters, such as asking the interviewer to instruct their RCF to install a television in their bedroom. The intent of the interview and the position of the interviewer was explained again in these cases.

## Conclusion

Undertaking PPIE in a secondary data analysis project was beneficial to many aspects of the project. Residents highlighted the importance of privacy and that the database be anonymised which emphasised the significance of anonymization of the database during its development. The researchers in turn allocated additional resources to ensure this was achieved. Residents aided with dissemination and knowledge transfer plans. Despite the perceived lack of scope for PPIE in a secondary data analysis project residents’ contribution was valuable. Their enthusiasm for the project and the research conducted in turn enthused the researchers to complete and publish the database and to conduct further research.

Undertaking PPIE requires resources; time, finances and expertise. Undertaking PPIE with vulnerable groups requires further dedication of these resources. The COVID-19 pandemic necessitated alternative plans and use of resources. Researchers completing PPIE and facing similar obstacles should forecast the resources required to effectively undertake PPIE and overcome these obstacles. Overcoming the obstacles presented to this study was achievable and worthwhile and demonstrates that with the appropriate resources, expertise and planning**,** the benefits of PPIE can be achieved with any group during unprecedented times.

## Supplementary Information

**Additional file 1: Table 1**. GRIPP2 long form.

**Additional file 2**. The Project Interview Schedule.

## Data Availability

The datasets (interview notes) generated and analysed during the current study are not publicly available due privacy of residents and conditions of ethical approval for this study. The database developed as part of the LENS project, upon which the PPIE is based is the Database of Statutory Notifications from Social Care in Ireland, 2013–2019 (Open Access). Available at: https://www.hiqa.ie/areas-we-work/Database-of-Statutory-Notifications
