# Supplementary material for: To summarise the approach to and findings of the PPIE undertaken as part of a programme of secondary research with a vulnerable, hard to reach population during the COVID-19 pandemic
Source: Res Involv Engagem. 2023 May 10;9:31. doi: 10.1186/s40900-023-00416-7 (PMC10171140; doi:10.1186/s40900-023-00416-7)
Supplement: Supplementary file 2 — Additional file 2. The Project Interview Schedule. [file 40900_2023_416_MOESM2_ESM.docx]

**Supplementary material: Interview schedule**

**
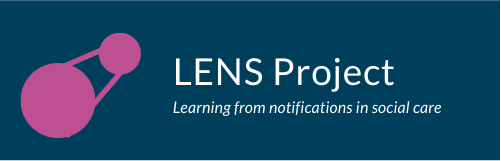
**

**Resident’s voice on statutory notification research**

**Interview Schedule Older Persons**

**Pre-amble**

Thanks for helping us with this research project. HIQA carries out inspections of nursing homes/care homes like yours. If something happens, like someone is injured or the electricity isn’t working HIQA is informed because this is important information about the care and welfare of residents. It is HIQA’s job to support homes like yours in providing safe care. There are 8 things that can happen that HIQA must be informed of within 3 days. These are, unexpected death, infectious disease, serious injury, unexplained absence of a resident, abuse of a resident, staff misconduct, professional review of staff member and the loss of electricity, heating, water or a fire or unplanned evacuation. In addition, all centres have to report to HIQA every quarter on matters such as the use of restraint, pressure sores and any thefts in a centre. We are working on a project to make better use of this information. All of the information about each event has been gathered together into one large dataset. This dataset will be refined, all personal information will be taken out and it will be made available to the public/researchers. Researchers will be able to use this information to answer questions that will help improve the quality and the safety of care facilities like yours.

**Descriptive and determinants**

Currently HIQA are notified about deaths, outbreak of an infectious disease, serious injury, unexplained absence, any form of abuse, staff misconduct, staff member under professional review, fire, loss of power, heat or water or an unexplained evacuation.

Is this list appropriate?

Are any of these that you don’t think HIQA should be notified about?

Thinking about the things HIQA is currently notified about, are there any other types of events you think HIQA should be notified about?

Currently HIQA is notified of serious events within 3 day and less serious events every 3 months.

Do you think this is appropriate?

How much time should be allowed to elapse before HIQA should be notified?

Would you suggest any changes to this?

**FAIR Data**

What information, from the notifications that HIQA has already received, should be included in a dataset that we will be made available to the public/researchers?

Do you think it is a good idea to allow other people use the information?

Are you comfortable with the public/researchers having access to this dataset?

What data, from notifications that HIQA has, should be withheld from the public/researchers?

Can you see any benefits or negatives to it?

**Sharing good practice**

The notifications include information on what happened after the event. There are examples here of good things people did when dealing with the event. We want to make this good practice available to other homes so they can copy it. What areas should we focus on, for example relating to deaths, illness, injury, or any of the ones I’ve mentioned already?

Which ones are most important to you?

In your opinion which areas do you think need most improvement?

How should we present this material to researchers and HIQA employees?

How should we present this information to people working in homes?

Should we share this information with people living in homes as an example of the type of care they should expect to receive?

Thank you very much for your help with this research. Do you have any questions for me?
